# Chrysanthemum Cutting Productivity and Rooting Ability Are Improved by Grafting

**DOI:** 10.1155/2013/286328

**Published:** 2013-06-25

**Authors:** Jing Zhang, Sumei Chen, Ruixia Liu, Jiafu Jiang, Fadi Chen, Weimin Fang

**Affiliations:** College of Horticulture, Nanjing Agricultural University, Nanjing, Jiangsu 210095, China

## Abstract

Chrysanthemum has been commercially propagated by rooting of cuttings, whereas the quality will decline over multiple collections from a single plant. Therefore, we compared the vigour, rooting ability, and some physiological parameters between cuttings harvested from nongrafted “Jinba” (non-grafted cuttings) with those collected from grafted “Jinba” plants onto *Artemisia scoparia* as a rootstock (grafted cuttings). The yield, length, node number, stem diameter, fresh weight, and dry weight of the grafted cuttings were superior to the non-grafted cuttings. Also grafted cuttings “Jinba” rooted 1 day earlier, but showing enhanced rooting quality including number, length, diameter, and dry weight of roots, where compared to the non-grafted. The physiological parameters that indicated contents of soluble protein, peroxidase activity, soluble sugar, and starch, ratios of soluble sugar/nitrogen ratio, and carbohydrate/nitrogen (C/N), as well as contents of indole-3-acetic acid (IAA) and abscisic acid (ABA), and IAA/ABA ratio were significantly increased in the grafted cuttings. This suggested their important parts in mediating rooting ability. Results from this study showed that grafting improved productivity and rooting ability related to an altered physiology, which provide a means to meet the increasing demand.

## 1. Introduction

Vegetative propagation is commonly used to multiply elite individual in a number of horticultural and silvicultural plants [1], and particularly frequently in chrysanthemum (*Chrysanthemum grandiflorum*). Chrysanthemum is one of the major world ornamental species, and cuttings are normally taken from a stock plant, but their vigour of cuttings tends to decline when the cuttings were harvested repeatedly from the same stock plants. It is laborious to reestablish the stock plants to meet the need of cuttings; the development of techniques to extend the period for cuttings collection would therefore be of significant economic interests.

Unlike nongrafted plants (namely, a stock plant propagated by using cuttings), grafted plants using rootstock of higher vigor or tolerant to abiotic stress have been shown to lead to improvements in economic yield [2], end-use quality [3], water and nutrient uptake and use efficiency [4, 5] and abiotic stress tolerance [6–8] and increase synthesis of endogenous hormones [9].

Herbaceous species *Artemisia scoparia* is a useful rootstock to enhance the heat tolerance of chrysanthemum cultivars of “Yidalihong” and “Qiuyi” by decreasing membrane permeability and enhancing activities of superoxide dismutase, peroxidase, catalase, ascorbate peroxidase activity, and soluble protein content [10]. In present study, we compared the productivity and rooting ability of chrysanthemum cuttings obtained from nongrafted (nongrafted cuttings) and grafted cuttings onto *Artemisia scoparia* (grafted cuttings). In addition, we investigated the content of a number of physiological, metabolic, and hormonal components in these two kinds of cuttings. It aids to improve the yield and quality of cuttings of cut chrysanthemum.

## 2. Materials and Methods

### 2.1. Plant Materials and Growing Conditions

Chrysanthemum “Jinba” and grafted “Jinba” onto *A. scoparia* were maintained by the Chrysanthemum Germplasm Resource Preserving Centre, Nanjing Agricultural University, China. Both nongrafted and grafted stock plants at 6–8 leaf stage were cultured in common cultivation condition. In this cultivation, stock plants of the two type were pinched for only one time, after which three nodes from the base of the stem remained. As such three lateral shoots outgrew on each stock plant. The stock plants for cuttings harvest were fertilized with N : P : K (14.0 : 2.0 : 8.0) every two weeks and watered every two days. The four blocks of rooted cuttings and grafted plants were randomly plotted.

### 2.2. Collection and Productivity of Cuttings

Three batches of cuttings were collected the harvest times were referred to as H1 (June 17, 2011, 23 days after the initial pinching), H3 (July 27, 63 days), H5 (September 5, 103 days). At each harvest, the basal three nodes of the stock plant's lateral branches were left. Ten randomly selected stock plants per harvest were used for the assessment of biomass production, cutting yield, the length, node number, diameter (diameter of the uppermost node), and the fresh and dry weight of the cuttings.

### 2.3. Determination of Rooting Capacity

To determine the rooting capacity of the nongrafted and grafted cuttings, a sample of 150 cuttings per harvest (namely, H1, H2, and H5) of 6–8 cm long with three or four nodes was planted in trays filled with a 1 : 1 mixture of perlite and rice chaff ash. During rooting, the material was intermittently treated with mist irrigation during the daylight maintaining a relative humidity >70%. The H5 cuttings were given 3 h of supplementary incandescent lighting (11 p.m. till 2 a.m., 100lx) during the night. Five days after planting cuttings, ten cuttings per treatment were randomly sampled every day to determine the shortest days required for rooting; the day when ~20% of the cuttings showed visible roots was recorded as rooted [11]. In addition, 15 days after planting the cuttings, root length, diameter, number, and dry matter were measured based on the data collected from 10 randomly sampled cuttings. The test was performed in three replications.

### 2.4. Physiological Parameters

The basal leaves and stems from cuttings (H1, H3, and H5) were sampled, respectively, for physiological parameters investigation. Protein content was determined following the method of Bradford [12], employing bovine serum albumin as a standard. Peroxidase (POD) activity was assessed as described by Argandoña et al. [13]. The soluble sugar was determined following the methods of van Handel [14] and Paul et al. [15]. The total nitrogen content of the material was obtained using the Kjeldahl method [16], and the carbohydrate/nitrogen (C/N) ratio was obtained by dividing the soluble sugar plus starch by the total nitrogen contents [17]. The indole-3-acetic acid (IAA), abscisic acid (ABA), and gibberellic acid (GA) were quantified by applying protocols [18–20], and the hormone standards were purchased from Sigma (St. Louis, MO, USA).

### 2.5. Statistical Analysis

A one-way analysis of variance (ANOVA) was followed by the application of Duncan's multiple range test (*P* < 0.05) to assess whether treatment means differed statistically from one another using SPSS v13.0 software package (Chicago, IL, USA).

## 3. Results

### 3.1. The Cutting Productivity

The cutting productivity of the nongrafted and grafted stock chrysanthemums was presented in Table 1. The result showed that the grafted stock plants yielded 42, 150, and 83 cuttings in H1, H3, and H5, respectively, approximately 14%, 53%, and 15% more than did the nongrafted in respective harvest time. As for the cutting quality, that is, the mean length, node number, diameter, and fresh and dry matter of the cuttings, the stock plants performed better in H3 and then in H5 than in H1, irrespective of nongrafted or grafted. Moreover, the quality of the cuttings from grafted stock plants was overall better than that from nongrafted stock plants, except for H1.

### 3.2. Cutting Rooting Capacity

Rooting capacity of cuttings from nongrafted and grafted stock plants was shown in Table 2. In H1, H3, and H5, the cuttings obtained from grafted stock plants developed roots one day earlier than did those from the nongrafted ones, and the former showed increased maximum length, mean length, mean diameter, mean number, and mean dry matter of roots. This indicated that the grafting improves rooting capacity of Chrysanthemum cuttings. Among the three harvest times herein, the cuttings from grafted stock plants in H3 exhibited better rooting characteristics than the other treatments, other than mean root length and diameter shorter than that in H1.

### 3.3. Biochemical Aspects of Cutting Performance

**Figure 1:**
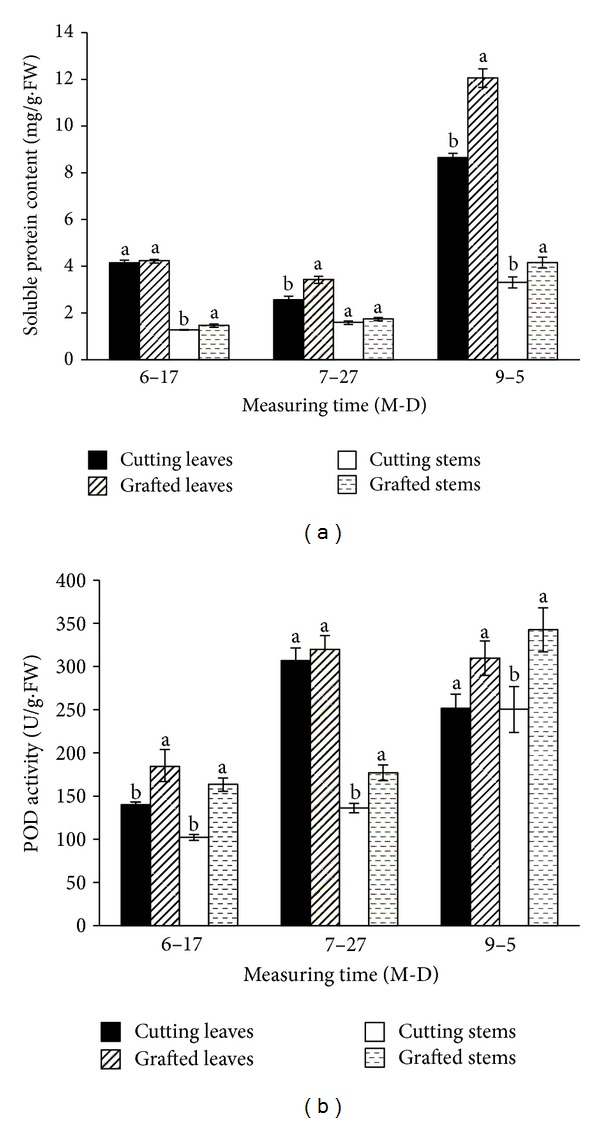
The contents of soluble protein and POD activity in the nongrafted and grafted cuttings. H1: cuttings harvested on June 17; H3: cuttings harvested on July 27; H5: cuttings harvested on September 5; means and standard errors calculated from three replicates. Significance (*P* < 0.05) indicated by lower case lettering ((a), (b)), derived using the Duncan's multiple range test.

**Figure 2:**
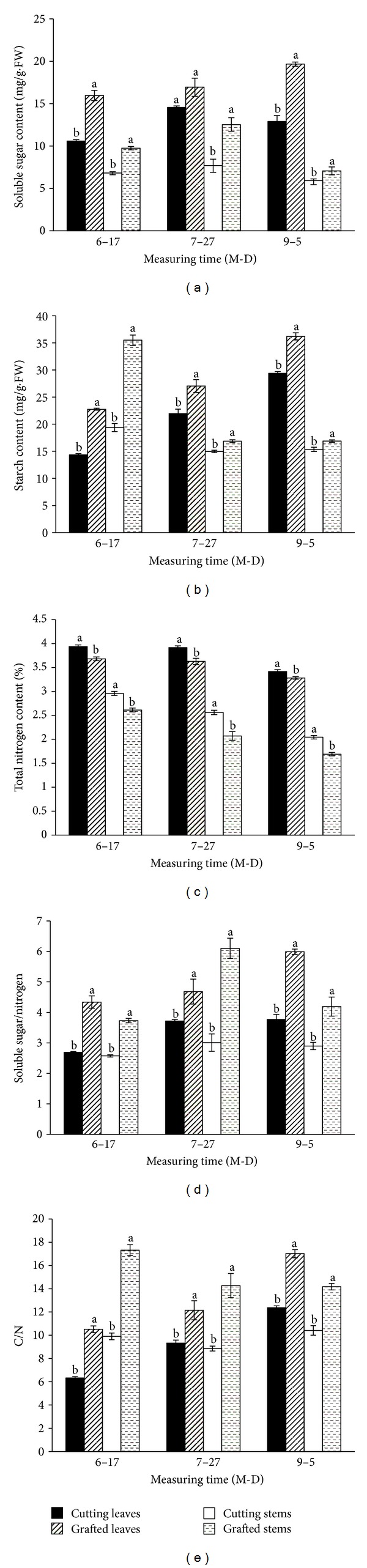
The contents of soluble sugar, starch, and total nitrogen, soluble sugar/nitrogen, and C/N in the nongrafted and grafted cuttings. H1, cuttings harvested on June 17; H3: cuttings harvested on July 27; H5: cuttings harvested on September 5; means and standard errors calculated from three replicates Significance (*P* < 0.05) indicated by lower case lettering ((a), (b)), derived using the Duncan's multiple range test.

Cuttings derived from grafted stock plants accumulated a greater level of soluble protein in their leaves and stems than their equivalents from nongrafted cuttings (Figure 1(a)). The difference was particularly notable in H5 cuttings, where soluble protein in the leaves and stems of grafted cuttings were, respectively, 39.4% and 25.8% higher than that of nongrafted cuttings. POD activity in the leaves and stems of cuttings derived from grafted cuttings tended to be higher than in their equivalents from nongrafted cuttings (Figure 1(b)). In all three samples (H1, H3, and H5), POD activity of stems in grafted cuttings was significantly higher, reaching 99.5% of the cutting seedlings in H1. Similarly, POD activity of leaves in the grafted cuttings was 24.1% higher than nongrafted cutting in H1. There were significant differences between the cuttings obtained from the two kinds of stock plants in soluble sugar and starch content of the leaves and stems (Figures 2(a) and 2(b)). Soluble sugar contents in leaves were higher than those in stems in all three batches. The overall performance of starch content cuttings was similar to the soluble sugar content. The influence of grafting on their total nitrogen content of the cuttings was illustrated in Figure 2(c). Generally, the level of total nitrogen content in grafted cuttings was lower than in nongrafted cuttings, irrespective of harvest time. The sugar/nitrogen and C/N ratios in the leaves and stems of the grafted cuttings were significantly higher than those in nongrafted cuttings (Figures 2(d) and 2(e)).

### 3.4. Phytohormone Content

**Figure 3:**
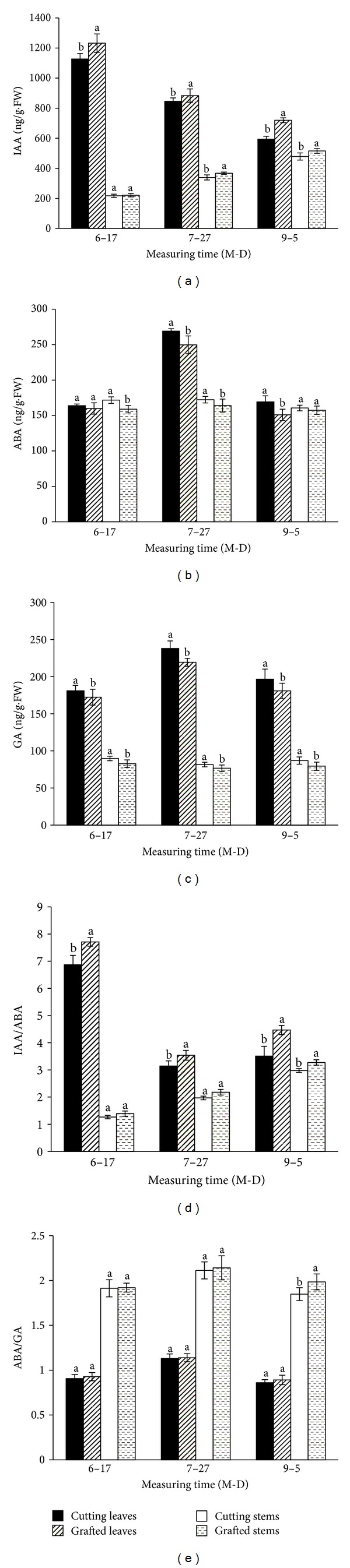
The contents of IAA, ABA and GA and the IAA/ABA and ABA/GA ratios in the nongrafted and grafted cuttings “Jinba”. H1: cuttings harvested on June 17; H3: cuttings harvested on July 27; H5: cuttings harvested on September 5; means and standard errors calculated from three replicates. Significance (*P* < 0.05) indicated by lower case lettering ((a), (b)), derived using the Duncan's multiple range test.

The IAA content in the leaves and stems of the grafted cuttings was significantly higher than that in nongrafted cuttings except that in stems harvested in H1 (Figure 3(a)). In contrast, a slight decrease of ABA and GA contents in the grafted cuttings was observed in all treatments (Figures 3(b) and 3(c)).  Figure 3(d) showed that IAA/ABA is in leaves of grafted cuttings in all three harvest times, and so observed in stems harvest in H5. In addition, there was no significance in ABA/GA ratio but for a slight increase in stems of grafted cuttings (Figure 3(e)).

## 4. Discussion

The well-established root system enjoyed by grafted plants was associated with an increased potential to generate biomass [21, 22]. Here, in chrysanthemum, cuttings taken from grafted stock plants were superior to those taken from nongrafted ones in term of cutting quality and production; also, we found that the grafted cuttings rooted earlier than the nongrafted, moreover, show improved root quality. In previous study we reported the vigorous root system of* A. scoparia* had a considerable influence on the uptake and translocation of water and nutrients and as such played an essential role in physiological processes of scion such as growth, signal transduction, and development [10]. Therefore, we ascribe the improved productivity and rooting capacity of grafted cutting to the vigorous root system of the rootstock, namely, *A. scoparia* in this study.

Chu et al. [23] had suggested that higher rooting ability had been related to an increase in synthesis of soluble protein during the root regeneration process. In the present study, the leaves and stems of grafted cuttings had higher level of soluble protein content than the nongrafted either (Figure 1(a)), suggesting grafting improving adventitious root formation was partially due to the elevated protein content in grafted cuttings. POD was a useful biochemical marker for analysis of rooting phases for correlation with tissue morphological changes [24]. POD activity in grafted cuttings in this study was higher than that in the nongrafted (Figure 1(b)) and similar to the result in eggplant [25]. Thus grafting might act to promote POD enzyme activities and result in mediating adventitious root development of the cuttings.

The carbohydrate and nitrogen statuses of the plant are known to be important determinants of adventitious root formation [1]. Rouphael et al. [4] noted that when mini-watermelon plants were grafted onto a *Cucurbita maxima* Duchesne × *Cucurbita moschata* Duchesne hybrid rootstock grown under open field conditions, the leaf nitrogen content in grafted plant was higher than nongrafted plants. Yang et al. [17] had suggested that the sugar/nitrogen and C/N ratios were also influential on rooting ability. In the present study, the grafted cuttings had higher level of soluble sugar and starch contents, as well as both superior sugar/nitrogen and C/N ratios (Figure 2). As a result, it is possible that soluble sugar, starch, and nitrogen in leaves and stems of grafted cuttings synergistically played a positive role in rooting of cut chrysanthemum. 

Endogenous phytohormones were thought to be involved in the complex relationship between the rootstock and the scion [26]. The IAA and GA content of grafted cucumber plants were both higher than in the nongrafted ones [27]. In this study, the grafted cuttings had more IAA but less ABA and GA than the nongrafted cuttings (Figure 3), such similar result had been reported in cucumber [28]. Also the IAA/ABA ratio was significantly higher in the grafted cuttings, compared with in that the nongrafted cuttings, while there was no significant difference in the ABA/GA ratio (Figures 3(d) and 3(e)). Yang [11] emphasized that the level of IAA/ABA played an important part in the rooting ability of chrysanthemum, and the cuttings with higher IAA/ABA were easier to rooting. It inferred that higher IAA/ABA ratio in the grafted chrysanthemum contributed to the better rooting ability. Our results showed that the grafting affected the level of various endogenous phytohormones over an extended period in chrysanthemum, and thereby lead to high production of more vigorous cuttings.

In summary, when chrysanthemum scions are grafted onto an *A. scoparia *rootstock, the resulting plants produce more vigorous cuttings and rooting ability related to an altered physiology. The study has thereby validated the idea of grafting improving the productivity of cutting production in chrysanthemum.

## Figures and Tables

**Table 1:** The productivity of and quality of cuttings from non-grafted and grafted chrysanthemum “Jinba”.

Batch	Treatment	Yield (no. of cuttings per 10 plants)	Mean length (cm)	Mean node number	Mean diameter (mm)	Mean fresh matter (g)	Mean dry matter (g)
H1	Non-grafted	37	8.1 ± 0.40a	5.2 ± 0.19b	3.43 ± 0.091a	1.98 ± 0.137a	0.244 ± 0.022a
	Grafted	42	8.1 ± 0.36a	6.3 ± 0.29a	3.14 ± 0.054b	1.98 ± 0.127a	0.236 ± 0.012a
H3	Non-grafted	98	5.1 ± 0.26b	5.9 ± 0.23a	2.95 ± 0.043b	0.99 ± 0.040b	0.163 ± 0.019b
	Grafted	150	6.1 ± 0.22a	6.3 ± 0.19a	3.35 ± 0.029a	1.48 ± 0.072a	0.203 ± 0.028a
H5	Non-grafted	72	6.9 ± 0.28b	7.3 ± 0.21b	2.50 ± 0.054b	1.09 ± 0.061b	0.191 ± 0.012b
	Grafted	83	8.6 ± 0.49a	9.1 ± 0.35a	2.72 ± 0.058a	1.42 ± 0.088a	0.246 ± 0.016a

Significant differences of different treatments are compared in the same batch. H1: cuttings harvested on June 17; H3: cuttings harvested on July 27; H5: cuttings harvested on September 5; values (mean ± S.E.) labelled with a different letter suffix differ significantly from one another at the same time point according to Duncan's multiple range test (*P* < 0.05).

**Table 2:** The rooting characteristics of the non-grafted and grafted cuttings “Jinba”.

Batch	Treatment	Rooting formation days (d)	Maximum length (cm)	Mean length (cm)	Mean diameter (mm)	Mean number	Mean dry matter (mg)
H1	Non-grafted	8	4.5	3.42 ± 0.24a	0.556 ± 0.016b	36.0 ± 1.27b	9.7 ± 0.08b
	Grafted	7	4.8	4.02 ± 0.20a	0.627 ± 0.011a	40.8 ± 0.79a	10.9 ± 0.19a
H3	Non-grafted	7	5.2	2.79 ± 0.17b	0.451 ± 0.019a	39.5 ± 2.48b	12.7 ± 0.58b
	Grafted	6	5.8	3.63 ± 0.10a	0.500 ± 0.016a	47.7 ± 3.03a	13.3 ± 0.39a
H5	Non-grafted	8	3.2	2.90 ± 0.07a	0.352 ± 0.014b	31.3 ± 1.63b	10.0 ± 0.21b
	Grafted	7	4.5	3.09 ± 0.19a	0.397 ± 0.012a	37.2 ± 0.93a	11.0 ± 0.16a

H1: cuttings harvested on June 17; H3: cuttings harvested on July 27; H5: cuttings harvested on September 5; values (mean ± S.E.) labelled with a different letter suffix differ significantly from one another according to Duncan's multiple range test (*P* < 0.05).

## References

[1] Liao W-B, Xiao H-L, Zhang M-L (2010). Effect of nitric oxide and hydrogen peroxide on adventitious root development from cuttings of ground-cover chrysanthemum and associated biochemical changes. *Journal of Plant Growth Regulation*.

[2] Lee JM, Oda M (2003). Grafting of herbaceous vegetable and ornamental crops. *Horticultural Reviews*.

[3] Colla G, Rouphael Y, Cardarelli M (2008). Influence of grafting on yield and fruit quality of pepper (*Capsicum annuum* L.) grown under greenhouse conditions. *Acta Horticulturae*.

[4] Rouphael Y, Cardarelli M, Colla G, Rea E (2008). Yield, mineral composition, water relations, and water use efficiency of grafted mini-watermelon plants under deficit irrigation. *HortScience*.

[5] Colla G, Suãrez CMC, Cardarelli M, Rouphael Y (2010). Improving nitrogen use efficiency in melon by grafting. *HortScience*.

[6] Abdelmageed AHA, Gruda N (2009). Influence of grafting on growth, development and some physiological parameters of tomatoes under controlled heat stress conditions. *The European Journal of Horticultural Science*.

[7] Estañ MT, Martinez-Rodriguez MM, Perez-Alfocea F, Flowers TJ, Bolarin MC (2005). Grafting raises the salt tolerance of tomato through limiting the transport of sodium and chloride to the shoot. *Journal of Experimental Botany*.

[8] Rivero RM, Ruiz JM, Sánchez E, Romero L (2003). Does grafting provide tomato plants an advantage against H_2_O_2_ production under conditions of thermal shock?. *Physiologia Plantarum*.

[9] Dong HH, Niu YH, Li WJ, Zhang DM (2008). Effects of cotton rootstock on endogenous cytokinins and abscisic acid in xylem sap and leaves in relation to leaf senescence. *Journal of Experimental Botany*.

[10] Fang WM, Guo WM, Chen JY (2009). Effects of grafting on the improvement of heat tolerance and antioxidant abilities in leaves of chrysanthemum. *Acta Horticulturae Sinica*.

[11] Yang XM (2009). *Studies on the cutting propagation technology and rooting mechanism of chrysanthemum [Dissertation]*.

[12] Bradford MM (1976). A rapid and sensitive method for the quantitation of microgram quantities of protein utilizing the principle of protein dye binding. *Analytical Biochemistry*.

[13] Argandoña VH, Chaman M, Cardemil L, Muñoz O, Zúñiga GE, Corcuera LJ (2001). Ethylene production and peroxidase activity in aphid-infested barley. *Journal of Chemical Ecology*.

[14] van Handel E (1968). Direct microdetermination of sucrose. *Analytical Biochemistry*.

[15] Paul MJ, Driscoll SP, Lawlor DW (1991). The effect of cooling on photosynthesis, amounts of carbohydrate and assimilate export in sunflower. *Journal of Experimental Botany*.

[16] Li HS, Sun Q, Zhao SJ, Zhang WH (2000). *Experiment Principle and Technique For Plant Physiology and Biochemistry*.

[17] Yang XM, Fang WM, Chen FD, Chen SM (2010). Cuttings rooting process and changes in carbohydrate, nitrogen and endogenous hormone levels during the rooting of two chrysanthemum cultivars. *Journal of Nanjing Agricultural University*.

[18] Davenport TL, Pearce DW, Rood SB (2001). Correlation of endogenous gibberellic acid with initiation of mango shoot growth. *Journal of Plant Growth and Regulation*.

[19] Takei K, Sakakibara H, Taniguchi M, Sugiyama T (2001). Nitrogen-dependent accumulation of cytokinins in root and the translocation to leaf: implication of cytokinin species that induces gene expression of maize response regulator. *Plant and Cell Physiology*.

[20] Walch-Liu P, Neumann G, Bangerth F, Engels C (2000). Rapid effects of nitrogen form on leaf morphogenesis in tobacco. *Journal of Experimental Botany*.

[21] Salam MA, Masum ASMH, Chowdhury SS, Dhar M, Saddeque A, Islam MR (2002). Growth and yield of watermelon as influenced by grafting. *Journal of Biological Science*.

[22] Flores FB, Sanchez-Bell P, Estaén MT (2010). The effectiveness of grafting to improve tomato fruit quality. *Scientia Horticulturae*.

[23] Chu EP, Tavares AR, Kanashiro S, Giampaoli P, Yokota ES (2010). Effects of auxins on soluble carbohydrates, starch and soluble protein content in *Aechmea blanchetiana* (Bromeliaceae) cultured *in vitro*. *Scientia Horticulturae*.

[24] Rout GR (2006). Effect of auxins on adventitious root development from single node cuttings of *Camellia sinensis* (L.) Kuntze and associated biochemical changes. *Plant Growth Regulation*.

[25] Wei G-P, Yang L-F, Zhu Y-L, Chen G (2009). Changes in oxidative damage, antioxidant enzyme activities and polyamine contents in leaves of grafted and non-grafted eggplant seedlings under stress by excess of calcium nitrate. *Scientia Horticulturae*.

[26] Sorce C, Massai R, Picciarelli P, Lorenzi R (2002). Hormonal relationships in xylem sap of grafted and ungrafted *Prunus* rootstocks. *Scientia Horticulturae*.

[27] Kato T, Lou H (1989). Effect of rootstock on the yield, mineral nutrition and hormone level in xylem sap in eggplant. *Journal of Japanese Society For Horticultural Science*.

[28] Yang LF (2007). *Physiological and biochemical characteristics of salt tolerance in grafted cucumber using salt tolerant root stock [Ph.D Dissertation]*.

